# Supplementary figures and images for: Convergent Canonical Pathways in Autism Spectrum Disorder from Proteomic, Transcriptomic and DNA Methylation Data
Source: Int J Mol Sci. 2021 Oct 5;22(19):10757. doi: 10.3390/ijms221910757 (PMC8509728; doi:10.3390/ijms221910757)

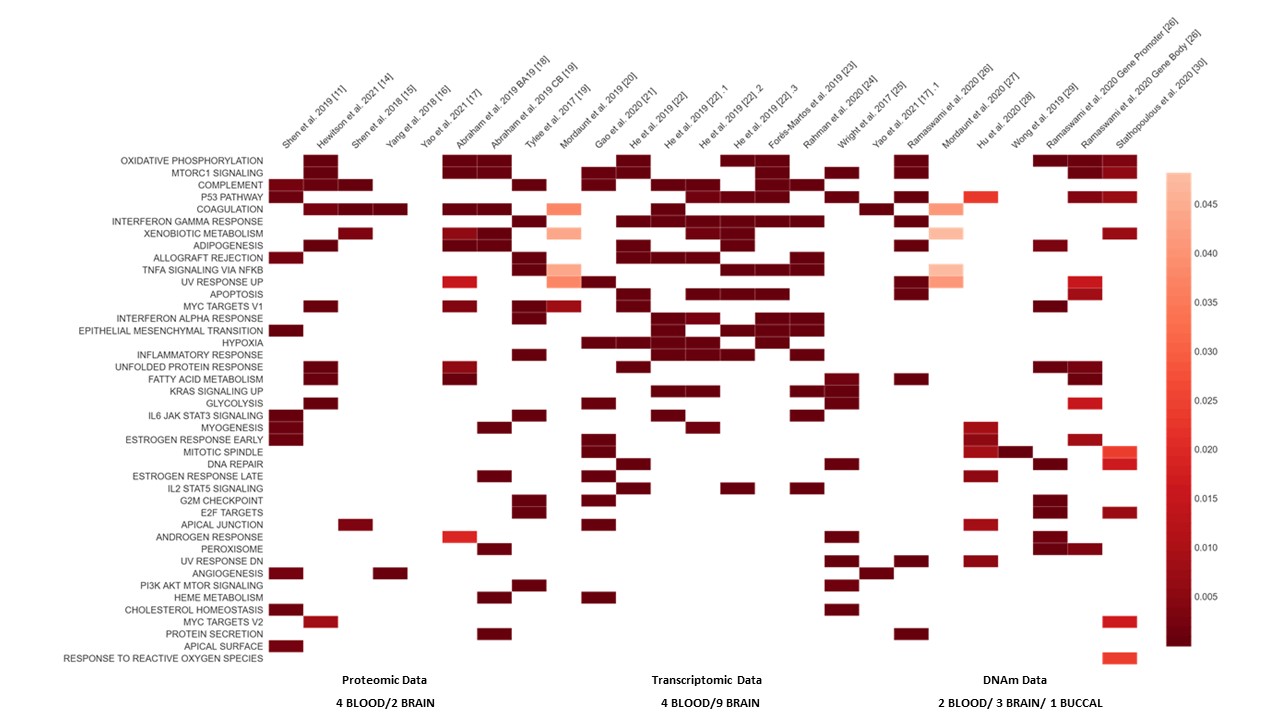

Supplement: Supplementary file 1 [file ijms-22-10757-s001.zip › Supplementary Fig.S1.jpg]

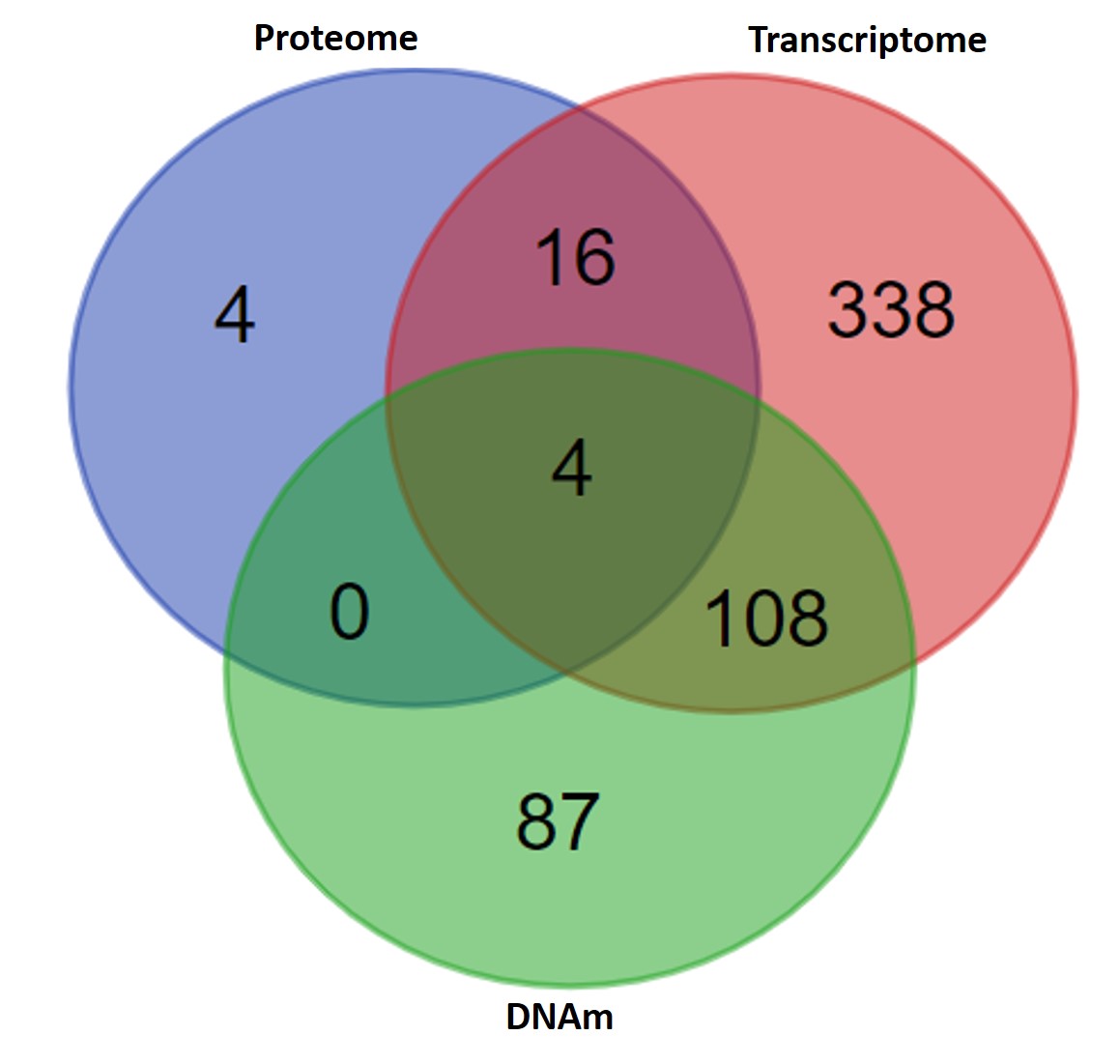

Supplement: Supplementary file 1 [file ijms-22-10757-s001.zip › Supplementary FigS3.jpg]

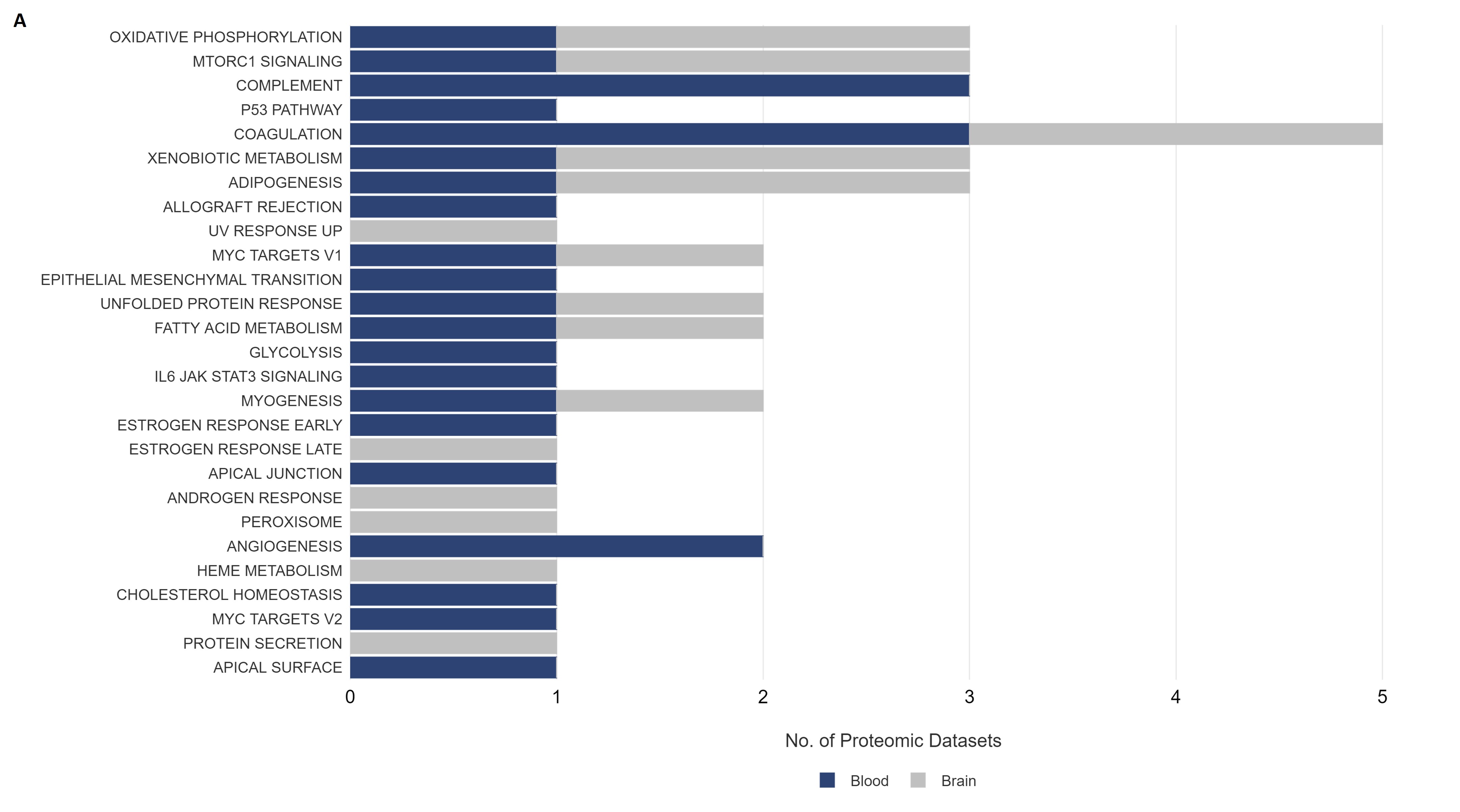

Supplement: Supplementary file 1 [file ijms-22-10757-s001.zip › Supplementary Figure S2A.jpg]

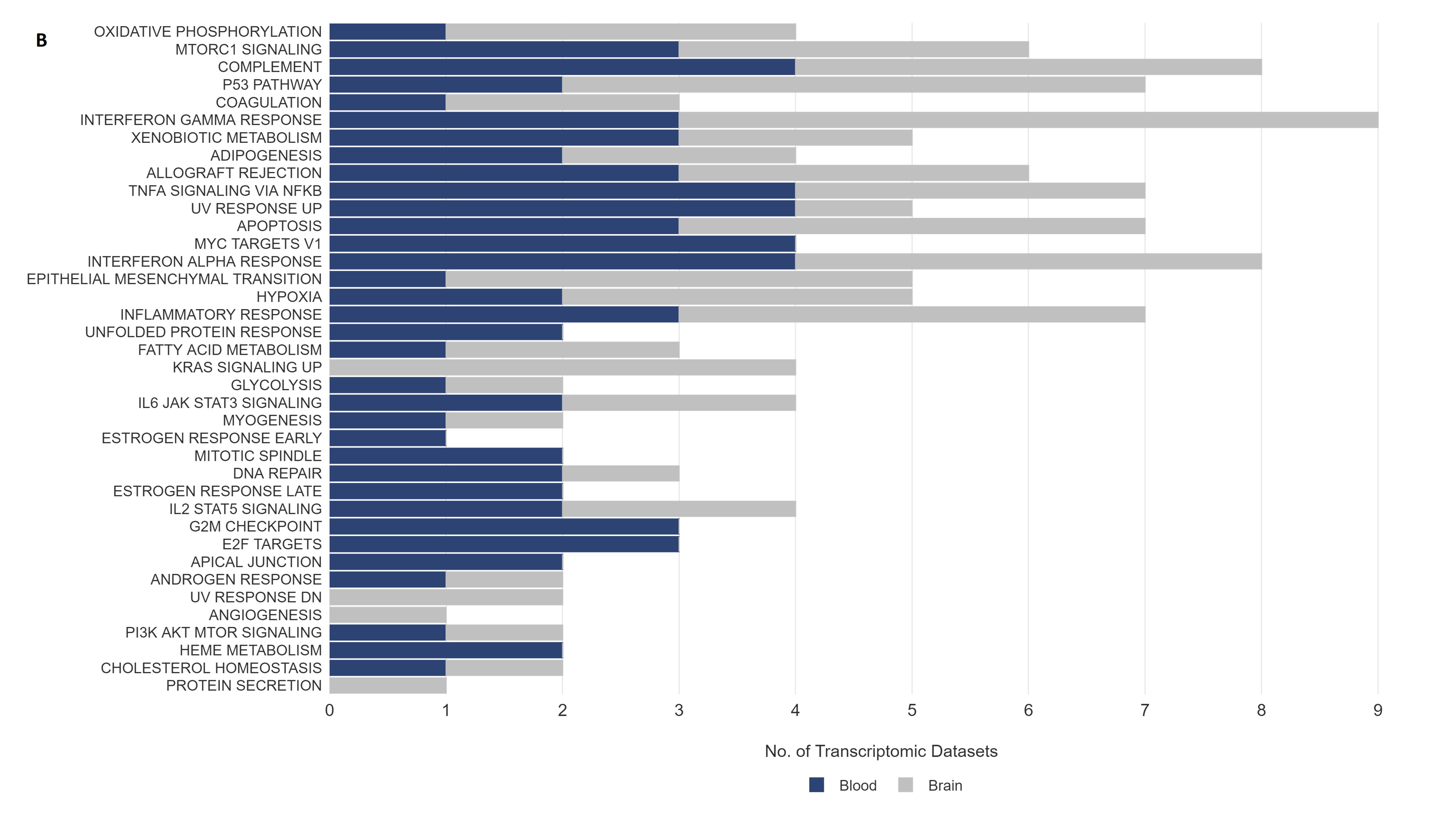

Supplement: Supplementary file 1 [file ijms-22-10757-s001.zip › Supplementary Figure S2B.jpg]

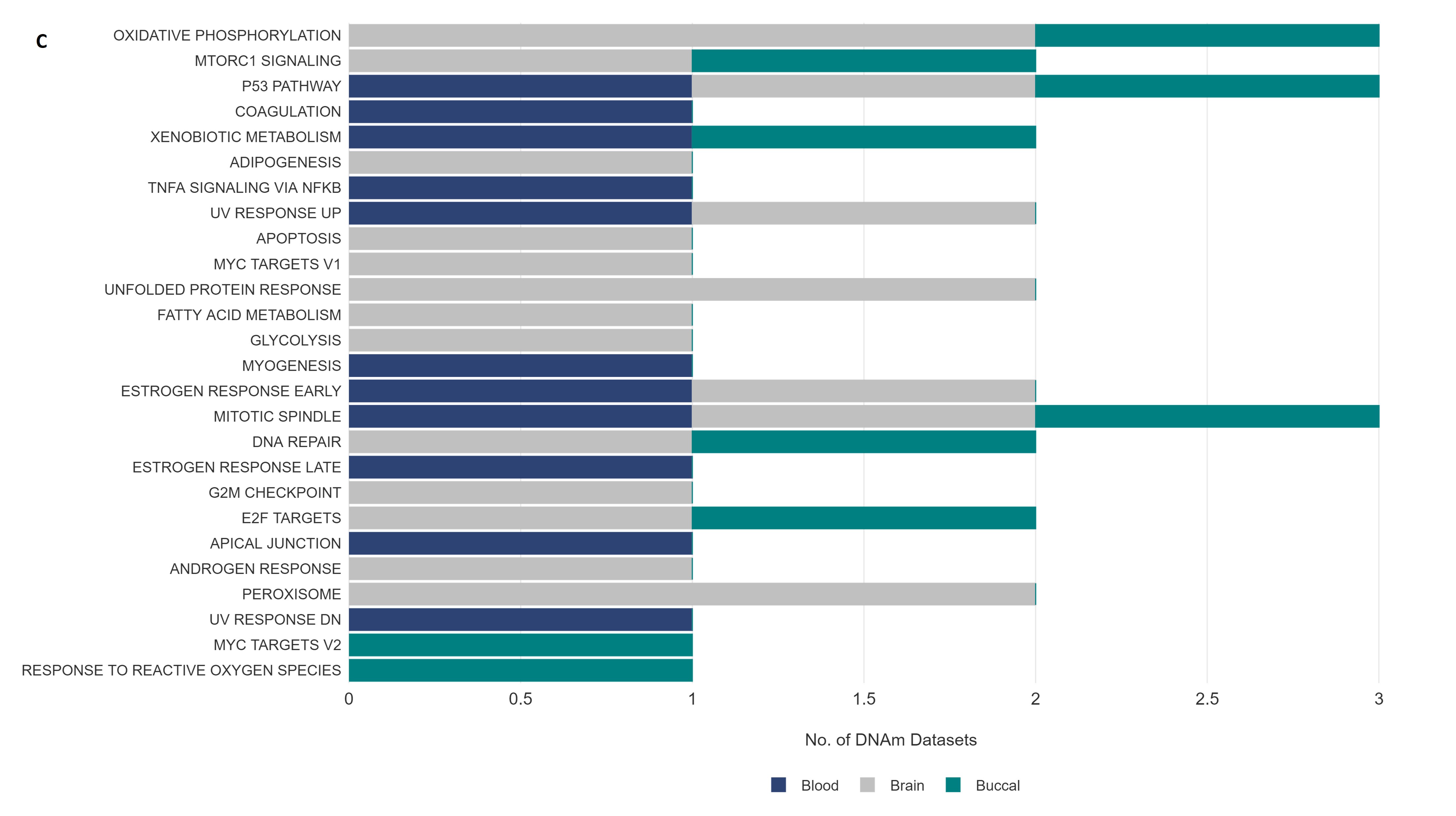

Supplement: Supplementary file 1 [file ijms-22-10757-s001.zip › Supplementary Figure S2C.jpg]
